# Bis(2,2′-bipyridine){ethyl 4′-[*N*-(4-carbamoylphen­yl)carbamo­yl]-2,2′-bi­pyridine-4-carboxyl­ate}ruthenium(II) bis­[hexa­fluorido­phosphate(V)]

**DOI:** 10.1107/S1600536809002360

**Published:** 2009-01-28

**Authors:** Masanari Hirahara, Shigeyuki Masaoka, Ken Sakai

**Affiliations:** a Department of Chemistry, Faculty of Science, Kyushu University, Hakozaki 6-10-1, Higashi-ku, Fukuoka 812-8581, Japan

## Abstract

In the title compound, [Ru(C_10_H_8_N_2_)_2_(C_21_H_18_N_4_O_4_)](PF_6_)_2_, the Ru^II^ complex cation reveals a slightly distorted octa­hedral coordination. The coordination bonds of the 4,4′-substituted bipyridyl donors [Ru—N = 2.038 (3) and 2.051 (3) Å] are shorter than those of the 2,2′-bipyridyl donors [Ru—N1 = 2.065 (3)–2.077 (3) Å], due to the electron-withdrawing effects of the substituents at the 4,4′-positions. The angles between the pyridyl planes of the three bipyridyl ligands are 1.5 (2), 6.3 (3) and 8.7 (2)°, respectively. The cations are connected by anions *via* N—H⋯F inter­actions.

## Related literature

For related literature, see: Gillaizeau-Gauthier *et al.* (2001 ▶); Ozawa & Sakai (2007 ▶); Ozawa *et al.* (2006 ▶, 2007 ▶); Sakai & Ozawa (2007 ▶); Sakai *et al.* (1993 ▶). For discussion of attractive inter­actions between negatively-charged atoms and alpha C atoms from heterocyclic rings, see: Schottel *et al.* (2008 ▶).
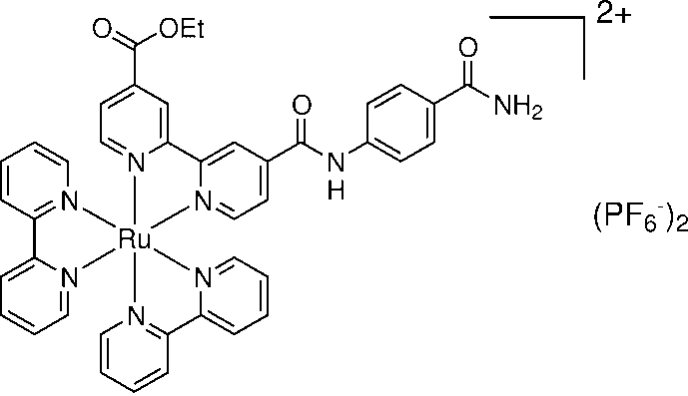


## Experimental

### 

#### Crystal data

[Ru(C_10_H_8_N_2_)_2_(C_21_H_18_N_4_O_4_)](PF_6_)_2_
*M* *_r_* = 1093.77
Monoclinic, 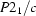

*a* = 18.400 (3) Å
*b* = 13.187 (2) Å
*c* = 18.863 (3) Å
β = 111.344 (2)°
*V* = 4262.9 (11) Å^3^
*Z* = 4
Mo *K*α radiation
μ = 0.55 mm^−1^
*T* = 100 **(s.u.?)** K
0.20 × 0.10 × 0.03 mm

#### Data collection

Bruker APEXII CCD area-detector diffractometer
Absorption correction: multi-scan (*SADABS*; Sheldrick, 1996 ▶) *T*_min_ = 0.717, *T*_max_ = 0.986
23329 measured reflections
9357 independent reflections
6405 reflections with *I* > 2σ(*I*)
*R*_int_ = 0.047

#### Refinement

*R*[*F* ^2^ > 2σ(*F* ^2^)] = 0.043
*wR*(*F* ^2^) = 0.113
*S* = 1.00
9357 reflections
614 parameters
H-atom parameters constrained
Δρ_max_ = 0.79 e Å^−3^
Δρ_min_ = −0.48 e Å^−3^

### 

Data collection: *APEX2* (Bruker, 2006 ▶); cell refinement: *APEX2*; data reduction: *SAINT* (Bruker, 2004 ▶); program(s) used to solve structure: *SHELXS97* (Sheldrick, 2008 ▶); program(s) used to refine structure: *SHELXL97* (Sheldrick, 2008 ▶); molecular graphics: *KENX* (Sakai, 2004 ▶); software used to prepare material for publication: *SHELXL97*, *TEXSAN* (Molecular Structure Corporation, 2001 ▶), *KENX* and *ORTEPII* (Johnson, 1976 ▶).

## Supplementary Material

Crystal structure: contains datablocks global, I. DOI: 10.1107/S1600536809002360/kp2198sup1.cif

Structure factors: contains datablocks I. DOI: 10.1107/S1600536809002360/kp2198Isup2.hkl

Additional supplementary materials:  crystallographic information; 3D view; checkCIF report

## Figures and Tables

**Table 1:** Selected bond lengths (Å)

Ru1—N1	2.077 (3)
Ru1—N2	2.070 (3)
Ru1—N3	2.076 (3)
Ru1—N4	2.065 (3)
Ru1—N5	2.051 (3)
Ru1—N6	2.038 (3)

**Table 2:** Hydrogen-bond geometry (Å, °)

*D*—H⋯*A*	*D*—H	H⋯*A*	*D*⋯*A*	*D*—H⋯*A*
N7—H7⋯F2^i^	0.86	2.34	3.181 (4)	168
N8—H8*B*⋯F10^i^	0.86	2.29	2.999 (5)	139

Symmetry code: (i) 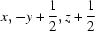.

**Fig. 1.**
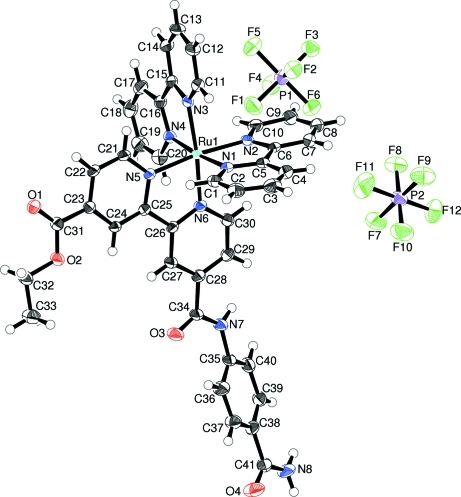
The molecular structure of (I) with the complex cation and anions showing the atom-labeling scheme. Displacement ellipsoids are drawn at the 50% probability level.

**Fig. 2.**
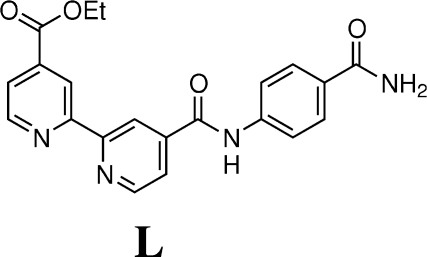
The ligand L.

## supplementary crystallographic information

### Comment

Continuous efforts have been made to elucidate the molecular catalysis of platinum(II) complexes in photochemical hydrogen production from water (Sakai *et al.*, 1993; Sakai & Ozawa, 2007; Ozawa *et al.*, 2007; Ozawa & Sakai, 2007). The results obtained so far suggest that destabilization of the HOMO, which generally corresponds to the filled Pt^II^*d**_z_*2 orbital, gives rise to the higher H_2_-evolving activity of the complexes (Sakai & Ozawa, 2007). It has also been ascertained that the amidate-bridged dinuclear platinum(II) complexes having a strong metal–metal interaction exhibit considerably higher H_2_-evolving activity in comparison with the mononuclear complexes, which has been attributed to their highly destabilized HOMOs arising from the anti-bonding couple of the filled Pt^II^*d**_z_*2 orbitals (Sakai & Ozawa, 2007). Moreover, the first effective model of a `photo-hydrogen-evolving' molecular device possessing both a light-harvesting capability and an H_2_-evolving activity was developed in our group (Ozawa & Sakai, 2006). Since this molecular device is made up of a photosensitizing tris(2,2'-bipyridine)ruthenium(II) derivative and a mononuclear (4-carbamoyl-4'-carboxy-2,2'-bipyridine)dichloroplatinum(II) fragment, it is important to develop an amidate-bridged diplatinum(II) complex tethered to tris(2,2'-bipyridine)ruthenium(II) photosensitizers. In order to develop such systems, tris(2,2'-bipyridine)ruthenium(II) derivatives having an uncoordinated amide group must be prepared as a synthetic precursor. The title compound has been prepared as one of such precursor compounds. The actual application of this complex ligand will be separately reported elsewhere.

In (I) (Fig. 1), the coordination  bonds from the 4,4'-substituted bipyridine ligand [Ru1—N5 = 2.051 (3) and Ru1—N6 = 2.038 (3) Å] are meaningfully shorter than those from the non-substituted 2,2'-bipyridine ligands [Ru1—N1 = 2.077 (3), Ru1—N2 = 2.070 (3), Ru1—N3 = 2.076 (3), and Ru1—N4 = 2.065 (3) Å] (Table 1). This can be interpreted in terms of the stronger backdonation in the former bonds due to the electron-withdrawing effects of the carbamoyl and ethoxycarbonyl groups in the 4,4'-substituted bipyridyl ligand.

All the three bipyridyl units do not form a planar geometry but the two pyridyl planes within each bipyridyl unit are tilted with each other as follows. Two pyridyl planes consisting of N1→C5 and N2→C10 are only slightly tilted at an angle of 1.5 (2)°. On the other hand, the dihedral angles between the N3→C15 and N4→C20 planes and that between the N5→C25 and N6→-C30 planes are somewhat larger: 6.3 (3) and 8.7 (2)°, respectively. The six-atom r.m.s. deviations given in the best-plane calculations for the N1→C5, N2→C10, N3→C15, N4→C20, N5→C25, and N6→C30 planes are 0.0053, 0.0038, 0.0079, 0.0019, 0.0181, and 0.0129, respectively.

On the other hand, the plane defined by atoms C31, O1, and O2 from the ethoxycarbonyl unit is slightly tilted with respect to the connecting pyridyl plane (N5→C25) at an angle of 4.5 (4)°. The carbamoyl plane defined with atoms C34, O3, and N7 is even more tilted with respect to the connecting pyridyl plane (N6→C30) at an angle of 15.1 (5)°. The aromatic plane consisting of atoms C35—C40 is tilted with respect to the above-mentioned carbamoyl unit (C34/O3/N7) at an angle of 26.6 (3)°, where the six-atom r.m.s. deviation given in the best-plane calculation for the C35–C40 plane was 0.0056. The C35–C40 plane is also tilted with regard to the terminal carbamoyl unit (C41/O4/N8) at an angle of 7.9 (2)°.

The crystal packing is stabilized with van der Waals interactions with contributions of the hydrogen bonds formed between the F atoms of PF_6_^-^ and the N—H units of carbamoyl groups (Table 2). Short intermolecular contacts [F4—C11 = 2.965 (4) Å and F3—C10 = 2.946 (5) Å] may be assigned as relatively weak hydrogen bonds. The other two short intermolecular contacts [O4—C1 = 2.974 (6) Å and O4—C26 = 3.010 (5) Å] may be due to attractive interactions between negatively-charged atoms and alpha C atoms from heterocyclic rings (Schottel *et al.*, 2008).

### Experimental

As described below, the ligand *L* was synthesized in three steps and was finally reacted with *cis*-RuCl_2_(bpy)_2_.2H_2_O to give the final product (I).

First, 4,4'-diethoxycarbonyl-2,2'-bipyridine was prepared according to the literature (Gillaizeau-Gauthier *et al.*, 2001).

Next, 4-carboxy-4'-ethoxycarbonyl-2,2'-bipyridine monohydrate  was prepared from the partial hydrolysis of 4,4'-diethoxycarbonyl-2,2'-bipyridine as follows. To a solution of 4,4'-diethoxycarbonyl-2,2'-bipyridine (1.50 g, 5.0 mmol) in absolute dichloromethane (200 ml) was dropwisely added a solution of potassium hydroxide (0.23 g, 5.00 mmol) in ethanol (50 ml) at 273 K over 1 h. This procedure was carried out under Ar atmosphere. The reaction mixture was further stirred for 1 d, during which the temperature of the solution was gradually raised to room temperature. The colourless solid precipitated was collected by filtration and washed with ethyl acetate. The ethyl acetate washing was evaporated to dryness to collect the unreacted 4,4'-diethoxycarbonyl-2,2'-bipyridine (0.525 g, 35%). The colourless precipitate was re-dissolved in water and acidified by 1 N hydrochloric acid to give the product as a colourless solid, which was collected by filtration and dried *in vacuo* (yield 0.812 g, 60%). Anal. Calcd for C_14_N_2_H_14_O_5_: C, 57.92; H, 4.86; N, 9.65. Found: C, 57.27; H, 4.68; N, 9.67. ^1^H NMR (300.27 MHz, d-DMSO): δ 1.38 (t, 3H, J = 7.0 Hz), 4.42 (q, 2H, J = 7.0 Hz), 7.93 (m, 2H), 8.84 (s, 2H), 8.93 (t, 2H, J = 4.6 Hz), 13.84 (s, 1H).

As the final step in the synthesis of ligand *L*, 4-carboxy-4'-ethoxycarbonyl-2,2'-bipyridine monohydrate (397 mg, 1.46 mmol), 1-ethyl-3-(3-dimethylaminopropyl) carbodiimide hydrochloride (EDC.HCl, 337 mg, 1.76 mmol) and 1-hydroxybenzotriazole (HOBT.H_2_O, 280 mg, 1.77 mmol) were dissolved in DMF (dimethylformamide) (40 ml). To a solution of 4-aminobenzamide (235 mg, 1.72 mmol) and *N*-methylmorpholine (0.3 ml) in DMF (20 ml) was dropwisely added the former solution at 273 K over 20 min. The reaction mixture was further stirred for 1 d in Ar, during which the temperature of the mixture was gradually raised to room temperature. The reaction mixture was then evaporated to a total volume of 5 ml followed by addition of water (200 ml). The white solid precipitated was collected by filtration and washed with water (20 ml), with an aqueous 5% NaHCO_3_ solution (20 ml), with an aqueous 5% citric acid solution (20 ml), and finally with water (20 ml). The white solid was dried *in vacuo* (yield 208 mg, 36.5%). The washing from the aqueous 5% NaHCO_3_ solution was acidified by HCl to give the unreacted starting bpy derivative (147.3 mg 37.1%). Anal. Calcd for *L*, C_21_H_18_N_4_O_4_: C, 64.61; H, 4.65; N, 14.35. Found: C, 64.17; H, 4.84; N, 13.89. ^1^H NMR (300.27 MHz, d-DMSO): δ 1.39 (t, 3H), 4.42 (q, 2H), 7.31 (s, broad), 7.87 (s, broad), 7.90 (s, 4H), 7.96 (d, 1H), 8.00 (d, 1H), 8.89 (d, 2H), 8.97 (t, 2H), 10.96 (s, 1H).

Compound (I) [RuL(bpy)_2_](PF_6_)_2_ was prepared as follows. A solution of ligand *L* (0.396 g, 1.02 mmol) and *cis*-RuCl_2_(bpy)_2_.2H_2_O (0.545, 1.05 mmol) in ethanol (150 ml) was refluxed for 12 h followed by filtration for the removal of insoluble materials. The filtrate was evaporated to dryness. The residue was redissolved in water (2–3 ml) followed by filtration for the removal of insoluble materials. To the filtrated was added an aqueous saturated NH_4_PF_6_ solution (2 ml). The dark red solid precipitated was collected by filtration and washed with a minimum amount of cold water. The crude product (1.08 g) was recrystallized twice from a 1:1 mixture of ethanol and water (yield, 0.60 g, 55%). Anal. Calcd for [RuL(bpy)_2_](PF_6_)_2_, C_41_H_34_N_8_O_4_RuP_2_F_12_: C, 45.02; H, 3.13; N, 10.24. Found: C, 44.95; H, 3.25; N, 10.18. ^1^H NMR (300.27 MHz, CD3CN): δ 1.40 (t, 3H, J = 7.0 Hz), 4.46 (q, 2H, J = 7.0 Hz), 5.98 (s, broad, 1H), 6.74 (s, broad, 1H) 7.42 (m, 4H), 7.71 (t, 4H, J = 5.5 Hz), 7.84 (m, 2H), 7.88 (s, 4H), 7.95 (d, 1H, J = 5.8 Hz), 7.96 (d, 1H, J = 5.8 Hz), 8.09 (m, 4H), 8.52 (d, 4H, J = 7.7 Hz), 9.03 (s, 2H), 9.10 (s, 2H), 9.30 (s, 1H). ESI-TOF MS (m/*z*): 402 ([RuL(bpy)_2_]^2+^), 949 ({[RuL(bpy)_2_](PF_6_)}^+^).

### Refinement

All H atoms were placed in idealized positions (methyl C—H = 0.96 Å, methylene C—H = 0.97 Å, aromatic C—H = 0.95 Å, and amide N—H = 0.86 Å), and included in the refinement in a riding-model approximation, with *U*_iso_(H) = 1.5Ueq(methyl C) and *U*_iso_(H) = 1.2Ueq(methylene C, aromatic C, and amide N). In the final difference Fourier map, the highest peak was located 0.88 Å from atom Ru1. The deepest hole was located 0.47 Å from atom P1.

### Crystal data

**Table:**

[Ru(C_10_H_8_N_2_)_2_(C_21_H_18_N_4_O_4_)](PF_6_)_2_	*F*(000) = 2200
*M**_r_* = 1093.77	*D*_x_ = 1.704 Mg m^−^^3^
Monoclinic, *P*2_1_/*c*	Mo *K*α radiation, λ = 0.71073 Å
Hall symbol: -P 2ybc	Cell parameters from 7706 reflections
*a* = 18.400 (3) Å	θ = 2.2–27.5°
*b* = 13.187 (2) Å	µ = 0.55 mm^−^^1^
*c* = 18.863 (3) Å	*T* = 100 K
β = 111.344 (2)°	Block, red
*V* = 4262.9 (11) Å^3^	0.2 × 0.1 × 0.03 mm
*Z* = 4	

### Data collection

**Table:**

Bruker SMART APEXII CCD area-detector diffractometer	9357 independent reflections
Radiation source: rotating anode with a mirror focusing unit	6405 reflections with *I* > 2σ(*I*)
graphite	*R*_int_ = 0.047
φ and ω scans	θ_max_ = 27.1°, θ_min_ = 2.2°
Absorption correction: empirical (using intensity measurements) (*SADABS*; Sheldrick, 1996)	*h* = −23→19
*T*_min_ = 0.717, *T*_max_ = 0.986	*k* = −16→16
23329 measured reflections	*l* = −24→24

### Refinement

**Table:**

Refinement on *F*^2^	Primary atom site location: structure-invariant direct methods
Least-squares matrix: full	Secondary atom site location: difference Fourier map
*R*[*F*^2^ > 2σ(*F*^2^)] = 0.043	Hydrogen site location: inferred from neighbouring sites
*wR*(*F*^2^) = 0.113	H-atom parameters constrained
*S* = 1.00	*w* = 1/[σ^2^(*F*_o_^2^) + (0.0524*P*)^2^ + 2.5961*P*] where *P* = (*F*_o_^2^ + 2*F*_c_^2^)/3
9357 reflections	(Δ/σ)_max_ = 0.001
614 parameters	Δρ_max_ = 0.79 e Å^−^^3^
0 restraints	Δρ_min_ = −0.48 e Å^−^^3^

### Special details

**Table:**

Experimental. The first 50 frames were rescanned at the end of data collection to evaluate any possible decay phenomenon. Since it was judged to be negligible, no decay correction was applied to the data.
Geometry. All e.s.d.'s (except the e.s.d. in the dihedral angle between two l.s. planes) are estimated using the full covariance matrix. The cell e.s.d.'s are taken into account individually in the estimation of e.s.d.'s in distances, angles and torsion angles; correlations between e.s.d.'s in cell parameters are only used when they are defined by crystal symmetry. An approximate (isotropic) treatment of cell e.s.d.'s is used for estimating e.s.d.'s involving l.s. planes.Least-squares planes (*x*,*y*,*z* in crystal coordinates) and deviations from them (* indicates atom used to define plane)- 12.7740 (0.0188) *x* - 4.2078 (0.0170) *y* + 16.1006 (0.0145) *z* = 0.0050 (0.0090)* -0.0058 (0.0021) N1 * 0.0092 (0.0023) C1 * -0.0056 (0.0026) C2 * -0.0010 (0.0026) C3 * 0.0042 (0.0024) C4 * -0.0009 (0.0022) C5Rms deviation of fitted atoms = 0.0053-12.4144 (0.0196) *x* - 4.3031 (0.0184) *y* + 16.2635 (0.0138) *z* = 0.1580 (0.0125)Angle to previous plane (with approximate e.s.d.) = 1.54 (1/5)* 0.0017 (0.0022) N2 * -0.0044 (0.0023) C6 * 0.0017 (0.0025) C7 * 0.0035 (0.0027) C8 * -0.0061 (0.0027) C9 * 0.0036 (0.0025) C10Rms deviation of fitted atoms = 0.00388.6305 (0.0228) *x* + 5.1793 (0.0182) *y* + 10.6773 (0.0218) *z* = 5.3175 (0.0062)Angle to previous plane (with approximate e.s.d.) = 85.47 (0.09)* 0.0065 (0.0022) N3 * 0.0045 (0.0024) C11 * -0.0115 (0.0026) C12 * 0.0077 (0.0027) C13 * 0.0033 (0.0025) C14 * -0.0105 (0.0023) C15Rms deviation of fitted atoms = 0.00799.8523 (0.0240) *x* + 4.0319 (0.0188) *y* + 10.1554 (0.0254) *z* = 5.7461 (0.0068)Angle to previous plane (with approximate e.s.d.) = 6.28 (0.26)* 0.0018 (0.0023) N4 * -0.0026 (0.0024) C16 * 0.0007 (0.0026) C17 * 0.0019 (0.0028) C18 * -0.0026 (0.0028) C19 * 0.0009 (0.0026) C20Rms deviation of fitted atoms = 0.00195.2914 (0.0916) *x* - 12.4015 (0.0303) *y* + 1.2118 (0.0485) *z* = 1.7169 (0.0084)Angle to previous plane (with approximate e.s.d.) = 89.47 (0.22)* 0.0000 (0.0000) C31 * 0.0000 (0.0000) O1 * 0.0000 (0.0000) O2Rms deviation of fitted atoms = 0.00006.6694 (0.0231) *x* - 12.0776 (0.0072) *y* + 0.5442 (0.0266) *z* = 1.6902 (0.0034)Angle to previous plane (with approximate e.s.d.) = 4.54 (0.40)* -0.0271 (0.0023) N5 * 0.0077 (0.0025) C21 * 0.0156 (0.0025) C22 * -0.0197 (0.0024) C23 * 0.0005 (0.0024) C24 * 0.0229 (0.0023) C25Rms deviation of fitted atoms = 0.01819.0772 (0.0232) *x* - 11.3634 (0.0101) *y* - 1.3032 (0.0274) *z* = 1.6621 (0.0095)Angle to previous plane (with approximate e.s.d.) = 8.69 (0.16)* 0.0155 (0.0024) N6 * -0.0114 (0.0024) C26 * -0.0060 (0.0026) C27 * 0.0191 (0.0026) C28 * -0.0152 (0.0027) C29 * -0.0021 (0.0026) C30Rms deviation of fitted atoms = 0.01295.9317 (0.1164) *x* - 12.4552 (0.0320) *y* - 3.3218 (0.1161) *z* = 0.5112 (0.0477)Angle to previous plane (with approximate e.s.d.) = 15.10 (0.48)* 0.0000 (0.0000) C34 * 0.0000 (0.0000) O3 * 0.0000 (0.0000) N7Rms deviation of fitted atoms = 0.00002.1032 (0.0288) *x* + 12.8237 (0.0053) *y* + 2.7844 (0.0287) *z* = 0.1928 (0.0183)Angle to previous plane (with approximate e.s.d.) = 26.56 (0.29)* 0.0082 (0.0027) C35 * -0.0073 (0.0028) C36 * 0.0001 (0.0028) C37 * 0.0062 (0.0027) C38 * -0.0054 (0.0027) C39 * -0.0018 (0.0027) C40Rms deviation of fitted atoms = 0.0056-0.9384 (0.1032) *x* - 13.1238 (0.0039) *y* - 1.1164 (0.1264) *z* = 1.0753 (0.0983)Angle to previous plane (with approximate e.s.d.) = 7.87 (0.23)* 0.0000 (0.0000) C41 * 0.0000 (0.0000) O4 * 0.0000 (0.0000) N8Rms deviation of fitted atoms = 0.00002.1032 (0.0288) *x* + 12.8237 (0.0053) *y* + 2.7844 (0.0287) *z* = 0.1928 (0.0183)Angle to previous plane (with approximate e.s.d.) = 7.87 (0.23)* 0.0082 (0.0027) C35 * -0.0073 (0.0028) C36 * 0.0001 (0.0028) C37 * 0.0062 (0.0027) C38 * -0.0054 (0.0027) C39 * -0.0018 (0.0027) C40Rms deviation of fitted atoms = 0.00569.0772 (0.0232) *x* - 11.3634 (0.0101) *y* - 1.3032 (0.0274) *z* = 1.6621 (0.0095)Angle to previous plane (with approximate e.s.d.) = 40.76 (0.09)* 0.0155 (0.0024) N6 * -0.0114 (0.0024) C26 * -0.0060 (0.0026) C27 * 0.0191 (0.0026) C28 * -0.0152 (0.0027) C29 * -0.0021 (0.0026) C30Rms deviation of fitted atoms = 0.0129
Refinement. Refinement of *F*^2^ against ALL reflections. The weighted *R*-factor *wR* and goodness of fit *S* are based on *F*^2^, conventional *R*-factors *R* are based on *F*, with *F* set to zero for negative *F*^2^. The threshold expression of *F*^2^ > σ(*F*^2^) is used only for calculating *R*-factors(gt) *etc*. and is not relevant to the choice of reflections for refinement. *R*-factors based on *F*^2^ are statistically about twice as large as those based on *F*, and *R*- factors based on ALL data will be even larger.

### Fractional atomic coordinates and isotropic or equivalent isotropic displacement parameters (Å^2^)

**Table:**

	*x*	*y*	*z*	*U*_iso_*/*U*_eq_	
Ru1	0.294461 (16)	0.04801 (2)	0.250977 (15)	0.02158 (8)	
P1	0.35302 (6)	0.46905 (8)	0.15958 (6)	0.0353 (2)	
P2	0.29204 (6)	0.62723 (9)	0.45315 (6)	0.0387 (3)	
F1	0.26565 (15)	0.4258 (2)	0.12279 (15)	0.0540 (7)	
F2	0.32351 (14)	0.57383 (17)	0.11340 (13)	0.0438 (6)	
F3	0.43974 (13)	0.51275 (18)	0.19471 (13)	0.0428 (6)	
F4	0.38170 (15)	0.36573 (18)	0.20517 (13)	0.0478 (6)	
F5	0.37108 (15)	0.42579 (18)	0.08811 (13)	0.0496 (6)	
F6	0.33452 (15)	0.51482 (18)	0.22961 (13)	0.0472 (6)	
F7	0.26997 (18)	0.5185 (2)	0.47525 (18)	0.0704 (9)	
F8	0.37419 (15)	0.5834 (2)	0.45629 (16)	0.0562 (7)	
F9	0.31532 (17)	0.7367 (2)	0.43274 (16)	0.0643 (8)	
F10	0.20992 (15)	0.6720 (2)	0.44945 (17)	0.0688 (8)	
F11	0.25655 (19)	0.5986 (3)	0.36555 (15)	0.0821 (10)	
F12	0.32822 (17)	0.6553 (2)	0.54043 (14)	0.0601 (7)	
O1	−0.03074 (15)	−0.15596 (19)	−0.04502 (13)	0.0319 (6)	
O2	−0.06958 (14)	−0.16271 (19)	0.05549 (14)	0.0318 (6)	
O3	0.03822 (16)	−0.1340 (2)	0.41695 (15)	0.0451 (7)	
O4	−0.06524 (18)	−0.1397 (2)	0.73340 (17)	0.0510 (8)	
N1	0.24580 (17)	0.1913 (2)	0.24496 (16)	0.0242 (6)	
N2	0.38261 (16)	0.1246 (2)	0.33484 (16)	0.0261 (7)	
N3	0.35294 (16)	0.0746 (2)	0.17716 (15)	0.0232 (6)	
N4	0.35305 (16)	−0.0874 (2)	0.25818 (16)	0.0246 (6)	
N5	0.19777 (16)	−0.0206 (2)	0.17421 (15)	0.0231 (6)	
N6	0.23667 (16)	0.0047 (2)	0.31992 (15)	0.0240 (6)	
N7	0.14210 (19)	−0.1138 (2)	0.52667 (16)	0.0342 (8)	
H7	0.1919	−0.1061	0.5438	0.041*	
N8	0.0512 (2)	−0.1560 (3)	0.82715 (18)	0.0451 (9)	
H8A	0.0302	−0.1573	0.8611	0.054*	
H8B	0.1010	−0.1607	0.8405	0.054*	
C1	0.1751 (2)	0.2201 (3)	0.1973 (2)	0.0296 (8)	
H1	0.1448	0.1731	0.1623	0.036*	
C2	0.1449 (2)	0.3159 (3)	0.1975 (2)	0.0360 (9)	
H2	0.0961	0.3335	0.1626	0.043*	
C3	0.1892 (2)	0.3850 (3)	0.2510 (2)	0.0396 (10)	
H3	0.1702	0.4499	0.2528	0.048*	
C4	0.2621 (2)	0.3569 (3)	0.3018 (2)	0.0343 (9)	
H4	0.2922	0.4028	0.3381	0.041*	
C5	0.2899 (2)	0.2597 (3)	0.2982 (2)	0.0260 (8)	
C6	0.3667 (2)	0.2223 (3)	0.3481 (2)	0.0272 (8)	
C7	0.4208 (2)	0.2798 (3)	0.4051 (2)	0.0346 (9)	
H7A	0.4093	0.3462	0.4140	0.042*	
C8	0.4918 (2)	0.2374 (3)	0.4481 (2)	0.0409 (10)	
H8	0.5282	0.2752	0.4864	0.049*	
C9	0.5087 (2)	0.1396 (3)	0.4345 (2)	0.0402 (10)	
H9	0.5566	0.1106	0.4627	0.048*	
C10	0.4523 (2)	0.0848 (3)	0.3776 (2)	0.0328 (9)	
H10	0.4632	0.0182	0.3688	0.039*	
C11	0.3504 (2)	0.1599 (3)	0.1376 (2)	0.0277 (8)	
H11	0.3173	0.2119	0.1401	0.033*	
C12	0.3955 (2)	0.1735 (3)	0.0931 (2)	0.0339 (9)	
H12	0.3915	0.2329	0.0654	0.041*	
C13	0.4458 (2)	0.0983 (3)	0.0907 (2)	0.0344 (9)	
H13	0.4775	0.1068	0.0624	0.041*	
C14	0.4490 (2)	0.0100 (3)	0.1306 (2)	0.0303 (8)	
H14	0.4827	−0.0419	0.1291	0.036*	
C15	0.4019 (2)	−0.0014 (3)	0.17289 (19)	0.0247 (7)	
C16	0.3991 (2)	−0.0939 (3)	0.21569 (19)	0.0267 (8)	
C17	0.4383 (2)	−0.1823 (3)	0.2130 (2)	0.0325 (9)	
H17	0.4696	−0.1854	0.1840	0.039*	
C18	0.4307 (2)	−0.2657 (3)	0.2536 (2)	0.0376 (9)	
H18	0.4569	−0.3255	0.2523	0.045*	
C19	0.3840 (2)	−0.2599 (3)	0.2962 (2)	0.0379 (9)	
H19	0.3779	−0.3156	0.3237	0.045*	
C20	0.3464 (2)	−0.1700 (3)	0.2973 (2)	0.0324 (9)	
H20	0.3151	−0.1662	0.3263	0.039*	
C21	0.1834 (2)	−0.0348 (3)	0.09948 (19)	0.0260 (8)	
H21	0.2217	−0.0162	0.0806	0.031*	
C22	0.1148 (2)	−0.0756 (3)	0.04975 (19)	0.0264 (8)	
H22	0.1072	−0.0845	−0.0014	0.032*	
C23	0.05712 (19)	−0.1033 (2)	0.07734 (19)	0.0228 (7)	
C24	0.07228 (19)	−0.0931 (2)	0.15475 (18)	0.0217 (7)	
H24	0.0350	−0.1127	0.1747	0.026*	
C25	0.14324 (19)	−0.0536 (3)	0.20223 (18)	0.0220 (7)	
C26	0.16747 (19)	−0.0442 (3)	0.28556 (18)	0.0228 (7)	
C27	0.1254 (2)	−0.0832 (3)	0.3282 (2)	0.0276 (8)	
H27	0.0784	−0.1168	0.3040	0.033*	
C28	0.1542 (2)	−0.0714 (3)	0.40656 (19)	0.0271 (8)	
C29	0.2228 (2)	−0.0174 (3)	0.4403 (2)	0.0323 (9)	
H29	0.2419	−0.0054	0.4925	0.039*	
C30	0.2623 (2)	0.0180 (3)	0.3961 (2)	0.0303 (8)	
H30	0.3089	0.0528	0.4197	0.036*	
C31	−0.0182 (2)	−0.1441 (3)	0.0220 (2)	0.0260 (8)	
C32	−0.1427 (2)	−0.2112 (3)	0.0082 (2)	0.0359 (9)	
H32A	−0.1322	−0.2763	−0.0100	0.043*	
H32B	−0.1702	−0.1689	−0.0355	0.043*	
C33	−0.1909 (2)	−0.2249 (3)	0.0568 (2)	0.0429 (10)	
H33A	−0.1612	−0.2612	0.1022	0.064*	
H33B	−0.2372	−0.2627	0.0290	0.064*	
H33C	−0.2053	−0.1598	0.0702	0.064*	
C34	0.1056 (2)	−0.1108 (3)	0.4501 (2)	0.0315 (9)	
C35	0.1075 (2)	−0.1283 (3)	0.5818 (2)	0.0308 (8)	
C36	0.0286 (2)	−0.1128 (3)	0.5644 (2)	0.0378 (9)	
H36	−0.0040	−0.0978	0.5149	0.045*	
C37	−0.0014 (2)	−0.1198 (3)	0.6221 (2)	0.0348 (9)	
H37	−0.0544	−0.1085	0.6104	0.042*	
C38	0.0448 (2)	−0.1430 (3)	0.6962 (2)	0.0319 (9)	
C39	0.1243 (2)	−0.1606 (3)	0.7128 (2)	0.0348 (9)	
H39	0.1565	−0.1773	0.7622	0.042*	
C40	0.1555 (2)	−0.1530 (3)	0.6560 (2)	0.0335 (9)	
H40	0.2085	−0.1646	0.6675	0.040*	
C41	0.0065 (2)	−0.1465 (3)	0.7539 (2)	0.0341 (9)	

### Atomic displacement parameters (Å^2^)

**Table:**

	*U*^11^	*U*^22^	*U*^33^	*U*^12^	*U*^13^	*U*^23^
Ru1	0.01934 (14)	0.02957 (16)	0.01735 (14)	−0.00295 (12)	0.00850 (10)	−0.00124 (11)
P1	0.0359 (6)	0.0409 (6)	0.0276 (5)	−0.0005 (5)	0.0101 (4)	0.0018 (4)
P2	0.0341 (6)	0.0527 (7)	0.0279 (6)	−0.0078 (5)	0.0097 (5)	0.0066 (5)
F1	0.0469 (16)	0.0628 (17)	0.0498 (16)	−0.0160 (12)	0.0143 (13)	−0.0035 (12)
F2	0.0455 (15)	0.0457 (14)	0.0387 (14)	0.0057 (11)	0.0135 (12)	0.0049 (10)
F3	0.0342 (13)	0.0524 (15)	0.0399 (14)	0.0004 (11)	0.0113 (11)	0.0025 (11)
F4	0.0603 (17)	0.0414 (14)	0.0410 (14)	0.0030 (12)	0.0178 (13)	0.0071 (11)
F5	0.0636 (17)	0.0508 (16)	0.0392 (14)	0.0076 (12)	0.0245 (13)	−0.0018 (11)
F6	0.0551 (16)	0.0537 (15)	0.0400 (14)	−0.0018 (12)	0.0258 (13)	−0.0037 (11)
F7	0.085 (2)	0.0571 (18)	0.089 (2)	−0.0241 (15)	0.0552 (19)	−0.0047 (15)
F8	0.0436 (16)	0.0684 (18)	0.0612 (17)	0.0038 (13)	0.0246 (14)	0.0138 (14)
F9	0.071 (2)	0.0601 (18)	0.0712 (19)	0.0054 (14)	0.0375 (16)	0.0281 (15)
F10	0.0386 (16)	0.092 (2)	0.076 (2)	−0.0015 (15)	0.0206 (15)	0.0006 (17)
F11	0.076 (2)	0.119 (3)	0.0323 (15)	0.0066 (19)	−0.0030 (15)	−0.0111 (16)
F12	0.074 (2)	0.0638 (18)	0.0302 (13)	0.0033 (14)	0.0043 (13)	0.0000 (12)
O1	0.0321 (15)	0.0444 (16)	0.0177 (13)	−0.0067 (12)	0.0075 (11)	−0.0026 (11)
O2	0.0277 (14)	0.0437 (16)	0.0232 (13)	−0.0095 (11)	0.0083 (11)	−0.0018 (11)
O3	0.0375 (17)	0.075 (2)	0.0254 (15)	−0.0233 (15)	0.0148 (13)	−0.0040 (14)
O4	0.048 (2)	0.075 (2)	0.0376 (17)	0.0021 (16)	0.0254 (16)	0.0076 (15)
N1	0.0264 (16)	0.0279 (16)	0.0231 (15)	−0.0044 (12)	0.0149 (13)	−0.0010 (12)
N2	0.0201 (15)	0.0392 (18)	0.0212 (15)	−0.0069 (13)	0.0102 (13)	−0.0022 (12)
N3	0.0225 (15)	0.0300 (17)	0.0166 (14)	−0.0072 (12)	0.0066 (12)	−0.0024 (11)
N4	0.0213 (15)	0.0286 (16)	0.0228 (15)	−0.0019 (12)	0.0066 (12)	0.0000 (12)
N5	0.0227 (15)	0.0296 (17)	0.0181 (14)	−0.0035 (12)	0.0088 (12)	−0.0030 (11)
N6	0.0240 (16)	0.0301 (16)	0.0187 (15)	−0.0019 (12)	0.0088 (12)	0.0007 (12)
N7	0.0313 (18)	0.052 (2)	0.0209 (16)	−0.0144 (15)	0.0118 (14)	−0.0006 (14)
N8	0.047 (2)	0.072 (3)	0.0225 (18)	−0.0069 (18)	0.0205 (17)	−0.0020 (16)
C1	0.0241 (19)	0.039 (2)	0.0270 (19)	−0.0012 (16)	0.0113 (16)	0.0035 (16)
C2	0.030 (2)	0.041 (2)	0.039 (2)	0.0045 (18)	0.0141 (19)	0.0054 (18)
C3	0.042 (3)	0.036 (2)	0.049 (3)	0.0061 (18)	0.026 (2)	0.0044 (18)
C4	0.038 (2)	0.035 (2)	0.035 (2)	−0.0044 (17)	0.0192 (19)	−0.0051 (17)
C5	0.0273 (19)	0.031 (2)	0.0248 (19)	−0.0051 (15)	0.0161 (16)	−0.0002 (14)
C6	0.028 (2)	0.037 (2)	0.0233 (18)	−0.0071 (16)	0.0172 (16)	−0.0057 (15)
C7	0.033 (2)	0.043 (2)	0.032 (2)	−0.0116 (17)	0.0164 (18)	−0.0117 (17)
C8	0.029 (2)	0.065 (3)	0.029 (2)	−0.014 (2)	0.0116 (18)	−0.0170 (19)
C9	0.024 (2)	0.063 (3)	0.031 (2)	−0.0044 (19)	0.0069 (17)	−0.0087 (19)
C10	0.025 (2)	0.047 (2)	0.028 (2)	0.0003 (17)	0.0110 (16)	−0.0025 (17)
C11	0.029 (2)	0.029 (2)	0.0257 (19)	−0.0028 (15)	0.0111 (16)	−0.0026 (15)
C12	0.037 (2)	0.037 (2)	0.032 (2)	−0.0074 (17)	0.0170 (18)	0.0010 (16)
C13	0.034 (2)	0.044 (2)	0.031 (2)	−0.0093 (18)	0.0193 (18)	0.0002 (17)
C14	0.027 (2)	0.039 (2)	0.028 (2)	−0.0009 (16)	0.0133 (16)	−0.0057 (16)
C15	0.0215 (18)	0.032 (2)	0.0191 (17)	−0.0036 (15)	0.0059 (15)	−0.0048 (14)
C16	0.0234 (19)	0.036 (2)	0.0192 (18)	−0.0033 (15)	0.0057 (15)	−0.0044 (15)
C17	0.032 (2)	0.039 (2)	0.027 (2)	0.0024 (17)	0.0118 (17)	−0.0041 (16)
C18	0.041 (2)	0.033 (2)	0.035 (2)	0.0064 (18)	0.0092 (19)	−0.0026 (17)
C19	0.041 (2)	0.035 (2)	0.038 (2)	−0.0015 (18)	0.0141 (19)	0.0076 (17)
C20	0.032 (2)	0.034 (2)	0.031 (2)	0.0028 (16)	0.0105 (17)	0.0042 (16)
C21	0.0257 (18)	0.035 (2)	0.0215 (17)	−0.0024 (15)	0.0132 (15)	−0.0013 (14)
C22	0.029 (2)	0.035 (2)	0.0182 (17)	−0.0023 (15)	0.0119 (15)	−0.0032 (14)
C23	0.0239 (18)	0.0220 (18)	0.0227 (18)	0.0014 (14)	0.0087 (15)	0.0003 (13)
C24	0.0218 (18)	0.0244 (18)	0.0208 (17)	−0.0014 (14)	0.0102 (14)	0.0005 (13)
C25	0.0246 (17)	0.0242 (18)	0.0189 (16)	−0.0013 (14)	0.0099 (14)	−0.0013 (13)
C26	0.0219 (17)	0.0287 (19)	0.0190 (17)	−0.0008 (15)	0.0087 (14)	−0.0026 (14)
C27	0.0275 (19)	0.034 (2)	0.0238 (19)	−0.0088 (15)	0.0118 (16)	−0.0020 (15)
C28	0.030 (2)	0.033 (2)	0.0209 (18)	−0.0034 (15)	0.0115 (16)	0.0007 (14)
C29	0.037 (2)	0.043 (2)	0.0173 (18)	−0.0094 (17)	0.0108 (16)	−0.0038 (15)
C30	0.027 (2)	0.044 (2)	0.0201 (18)	−0.0108 (16)	0.0078 (15)	−0.0031 (15)
C31	0.0248 (19)	0.027 (2)	0.0241 (19)	−0.0011 (14)	0.0070 (15)	0.0018 (14)
C32	0.027 (2)	0.049 (3)	0.027 (2)	−0.0105 (17)	0.0040 (17)	0.0000 (17)
C33	0.036 (2)	0.055 (3)	0.040 (2)	−0.005 (2)	0.016 (2)	0.008 (2)
C34	0.032 (2)	0.042 (2)	0.0246 (19)	−0.0125 (17)	0.0154 (17)	−0.0040 (16)
C35	0.037 (2)	0.035 (2)	0.0250 (19)	−0.0136 (17)	0.0166 (17)	−0.0004 (15)
C36	0.040 (2)	0.051 (3)	0.025 (2)	−0.0049 (19)	0.0137 (18)	0.0051 (17)
C37	0.032 (2)	0.043 (2)	0.031 (2)	−0.0001 (17)	0.0130 (18)	0.0044 (17)
C38	0.044 (2)	0.033 (2)	0.0236 (19)	−0.0080 (17)	0.0173 (18)	−0.0026 (15)
C39	0.039 (2)	0.042 (2)	0.0223 (19)	−0.0121 (18)	0.0102 (18)	−0.0018 (16)
C40	0.033 (2)	0.039 (2)	0.027 (2)	−0.0105 (17)	0.0085 (17)	−0.0013 (16)
C41	0.039 (2)	0.037 (2)	0.028 (2)	−0.0061 (18)	0.0153 (18)	0.0008 (16)

### Geometric parameters (Å, °)

**Table:**

Ru1—N1	2.077 (3)	C9—C10	1.392 (5)
Ru1—N2	2.070 (3)	C9—H9	0.9300
Ru1—N3	2.076 (3)	C10—H10	0.9300
Ru1—N4	2.065 (3)	C11—C12	1.389 (5)
Ru1—N5	2.051 (3)	C11—H11	0.9300
Ru1—N6	2.038 (3)	C12—C13	1.369 (5)
P1—F4	1.595 (2)	C12—H12	0.9300
P1—F3	1.596 (2)	C13—C14	1.376 (5)
P1—F6	1.598 (2)	C13—H13	0.9300
P1—F5	1.606 (2)	C14—C15	1.384 (5)
P1—F1	1.606 (3)	C14—H14	0.9300
P1—F2	1.618 (2)	C15—C16	1.474 (5)
P2—F12	1.579 (3)	C16—C17	1.381 (5)
P2—F11	1.585 (3)	C17—C18	1.376 (5)
P2—F7	1.587 (3)	C17—H17	0.9300
P2—F9	1.592 (3)	C18—C19	1.375 (5)
P2—F8	1.599 (3)	C18—H18	0.9300
P2—F10	1.600 (3)	C19—C20	1.376 (5)
O1—C31	1.210 (4)	C19—H19	0.9300
O2—C31	1.337 (4)	C20—H20	0.9300
O2—C32	1.463 (4)	C21—C22	1.380 (5)
O3—C34	1.208 (4)	C21—H21	0.9300
O4—C41	1.237 (5)	C22—C23	1.389 (4)
N1—C1	1.338 (4)	C22—H22	0.9300
N1—C5	1.373 (4)	C23—C24	1.390 (4)
N2—C10	1.348 (5)	C23—C31	1.499 (5)
N2—C6	1.364 (5)	C24—C25	1.388 (4)
N3—C11	1.341 (4)	C24—H24	0.9300
N3—C15	1.369 (4)	C25—C26	1.475 (4)
N4—C20	1.345 (4)	C26—C27	1.401 (4)
N4—C16	1.364 (4)	C27—C28	1.386 (5)
N5—C21	1.349 (4)	C27—H27	0.9300
N5—C25	1.364 (4)	C28—C29	1.386 (5)
N6—C30	1.351 (4)	C28—C34	1.509 (5)
N6—C26	1.363 (4)	C29—C30	1.372 (5)
N7—C34	1.354 (5)	C29—H29	0.9300
N7—C35	1.415 (4)	C30—H30	0.9300
N7—H7	0.8600	C32—C33	1.502 (5)
N8—C41	1.332 (5)	C32—H32A	0.9700
N8—H8A	0.8600	C32—H32B	0.9700
N8—H8B	0.8600	C33—H33A	0.9600
C1—C2	1.381 (5)	C33—H33B	0.9600
C1—H1	0.9300	C33—H33C	0.9600
C2—C3	1.384 (6)	C35—C36	1.384 (5)
C2—H2	0.9300	C35—C40	1.394 (5)
C3—C4	1.384 (5)	C36—C37	1.391 (5)
C3—H3	0.9300	C36—H36	0.9300
C4—C5	1.392 (5)	C37—C38	1.380 (5)
C4—H4	0.9300	C37—H37	0.9300
C5—C6	1.468 (5)	C38—C39	1.399 (5)
C6—C7	1.394 (5)	C38—C41	1.497 (5)
C7—C8	1.380 (6)	C39—C40	1.392 (5)
C7—H7A	0.9300	C39—H39	0.9300
C8—C9	1.373 (6)	C40—H40	0.9300
C8—H8	0.9300		
			
N6—Ru1—N5	78.87 (11)	N3—C11—C12	122.5 (3)
N6—Ru1—N4	95.56 (11)	N3—C11—H11	118.8
N5—Ru1—N4	87.86 (11)	C12—C11—H11	118.8
N6—Ru1—N2	95.43 (11)	C13—C12—C11	119.1 (4)
N5—Ru1—N2	172.71 (11)	C13—C12—H12	120.5
N4—Ru1—N2	97.28 (11)	C11—C12—H12	120.5
N6—Ru1—N3	173.37 (11)	C12—C13—C14	119.4 (3)
N5—Ru1—N3	97.42 (11)	C12—C13—H13	120.3
N4—Ru1—N3	78.73 (11)	C14—C13—H13	120.3
N2—Ru1—N3	88.68 (10)	C13—C14—C15	119.7 (3)
N6—Ru1—N1	88.57 (11)	C13—C14—H14	120.2
N5—Ru1—N1	96.73 (11)	C15—C14—H14	120.2
N4—Ru1—N1	174.36 (11)	N3—C15—C14	121.2 (3)
N2—Ru1—N1	78.46 (12)	N3—C15—C16	114.8 (3)
N3—Ru1—N1	97.36 (11)	C14—C15—C16	124.0 (3)
F4—P1—F3	90.00 (14)	N4—C16—C17	121.2 (3)
F4—P1—F6	90.29 (13)	N4—C16—C15	115.0 (3)
F3—P1—F6	90.11 (13)	C17—C16—C15	123.8 (3)
F4—P1—F5	91.16 (13)	C18—C17—C16	119.6 (3)
F3—P1—F5	89.76 (14)	C18—C17—H17	120.2
F6—P1—F5	178.54 (15)	C16—C17—H17	120.2
F4—P1—F1	90.70 (14)	C19—C18—C17	119.5 (4)
F3—P1—F1	179.00 (15)	C19—C18—H18	120.3
F6—P1—F1	90.59 (14)	C17—C18—H18	120.3
F5—P1—F1	89.52 (14)	C18—C19—C20	118.7 (4)
F4—P1—F2	179.71 (15)	C18—C19—H19	120.6
F3—P1—F2	90.23 (13)	C20—C19—H19	120.6
F6—P1—F2	89.53 (13)	N4—C20—C19	122.9 (3)
F5—P1—F2	89.02 (13)	N4—C20—H20	118.5
F1—P1—F2	89.07 (14)	C19—C20—H20	118.5
F12—P2—F11	179.42 (19)	N5—C21—C22	123.1 (3)
F12—P2—F7	89.37 (16)	N5—C21—H21	118.4
F11—P2—F7	90.63 (19)	C22—C21—H21	118.4
F12—P2—F9	89.49 (16)	C21—C22—C23	118.9 (3)
F11—P2—F9	90.51 (18)	C21—C22—H22	120.6
F7—P2—F9	178.83 (19)	C23—C22—H22	120.6
F12—P2—F8	90.69 (16)	C22—C23—C24	118.6 (3)
F11—P2—F8	88.73 (17)	C22—C23—C31	118.2 (3)
F7—P2—F8	89.58 (16)	C24—C23—C31	123.2 (3)
F9—P2—F8	90.15 (15)	C25—C24—C23	119.7 (3)
F12—P2—F10	89.54 (16)	C25—C24—H24	120.1
F11—P2—F10	91.04 (17)	C23—C24—H24	120.1
F7—P2—F10	90.98 (17)	N5—C25—C24	121.4 (3)
F9—P2—F10	89.30 (16)	N5—C25—C26	114.0 (3)
F8—P2—F10	179.40 (17)	C24—C25—C26	124.6 (3)
C31—O2—C32	116.3 (3)	N6—C26—C27	121.1 (3)
C1—N1—C5	118.4 (3)	N6—C26—C25	114.5 (3)
C1—N1—Ru1	126.1 (2)	C27—C26—C25	124.4 (3)
C5—N1—Ru1	115.4 (2)	C28—C27—C26	119.6 (3)
C10—N2—C6	118.6 (3)	C28—C27—H27	120.2
C10—N2—Ru1	125.3 (3)	C26—C27—H27	120.2
C6—N2—Ru1	116.1 (2)	C27—C28—C29	118.6 (3)
C11—N3—C15	118.1 (3)	C27—C28—C34	118.0 (3)
C11—N3—Ru1	126.4 (2)	C29—C28—C34	123.2 (3)
C15—N3—Ru1	115.4 (2)	C30—C29—C28	119.4 (3)
C20—N4—C16	118.1 (3)	C30—C29—H29	120.3
C20—N4—Ru1	126.0 (2)	C28—C29—H29	120.3
C16—N4—Ru1	115.9 (2)	N6—C30—C29	123.0 (3)
C21—N5—C25	118.0 (3)	N6—C30—H30	118.5
C21—N5—Ru1	126.0 (2)	C29—C30—H30	118.5
C25—N5—Ru1	116.0 (2)	O1—C31—O2	124.8 (3)
C30—N6—C26	118.1 (3)	O1—C31—C23	123.4 (3)
C30—N6—Ru1	125.5 (2)	O2—C31—C23	111.8 (3)
C26—N6—Ru1	116.3 (2)	O2—C32—C33	107.3 (3)
C34—N7—C35	127.4 (3)	O2—C32—H32A	110.3
C34—N7—H7	116.3	C33—C32—H32A	110.3
C35—N7—H7	116.3	O2—C32—H32B	110.3
C41—N8—H8A	120.0	C33—C32—H32B	110.3
C41—N8—H8B	120.0	H32A—C32—H32B	108.5
H8A—N8—H8B	120.0	C32—C33—H33A	109.5
N1—C1—C2	123.5 (4)	C32—C33—H33B	109.5
N1—C1—H1	118.3	H33A—C33—H33B	109.5
C2—C1—H1	118.3	C32—C33—H33C	109.5
C1—C2—C3	118.3 (4)	H33A—C33—H33C	109.5
C1—C2—H2	120.9	H33B—C33—H33C	109.5
C3—C2—H2	120.9	O3—C34—N7	124.2 (3)
C2—C3—C4	119.6 (4)	O3—C34—C28	120.4 (3)
C2—C3—H3	120.2	N7—C34—C28	115.4 (3)
C4—C3—H3	120.2	C36—C35—C40	120.0 (3)
C3—C4—C5	119.6 (4)	C36—C35—N7	121.3 (3)
C3—C4—H4	120.2	C40—C35—N7	118.6 (3)
C5—C4—H4	120.2	C35—C36—C37	119.1 (4)
N1—C5—C4	120.7 (3)	C35—C36—H36	120.4
N1—C5—C6	115.0 (3)	C37—C36—H36	120.4
C4—C5—C6	124.3 (3)	C38—C37—C36	122.2 (4)
N2—C6—C7	120.9 (3)	C38—C37—H37	118.9
N2—C6—C5	114.8 (3)	C36—C37—H37	118.9
C7—C6—C5	124.2 (3)	C37—C38—C39	118.2 (3)
C8—C7—C6	119.4 (4)	C37—C38—C41	117.6 (4)
C8—C7—H7A	120.3	C39—C38—C41	124.2 (3)
C6—C7—H7A	120.3	C40—C39—C38	120.4 (4)
C9—C8—C7	120.0 (4)	C40—C39—H39	119.8
C9—C8—H8	120.0	C38—C39—H39	119.8
C7—C8—H8	120.0	C39—C40—C35	120.1 (4)
C8—C9—C10	118.4 (4)	C39—C40—H40	120.0
C8—C9—H9	120.8	C35—C40—H40	120.0
C10—C9—H9	120.8	O4—C41—N8	121.1 (4)
N2—C10—C9	122.6 (4)	O4—C41—C38	120.2 (4)
N2—C10—H10	118.7	N8—C41—C38	118.8 (4)
C9—C10—H10	118.7		
			
C5—N1—C1—C2	1.7 (5)	C21—N5—C25—C26	174.2 (3)
N1—C1—C2—C3	−1.7 (5)	C23—C24—C25—N5	2.6 (5)
C1—C2—C3—C4	0.7 (6)	C23—C24—C25—C26	−176.7 (3)
C2—C3—C4—C5	0.3 (5)	C30—N6—C26—C27	−2.4 (5)
C1—N1—C5—C4	−0.7 (5)	C30—N6—C26—C25	178.9 (3)
C1—N1—C5—C6	−180.0 (3)	N5—C25—C26—N6	6.1 (4)
C3—C4—C5—N1	−0.3 (5)	C24—C25—C26—N6	−174.6 (3)
C3—C4—C5—C6	178.9 (3)	N5—C25—C26—C27	−172.6 (3)
C10—N2—C6—C7	−0.5 (5)	C24—C25—C26—C27	6.7 (6)
C10—N2—C6—C5	179.2 (3)	N6—C26—C27—C28	0.4 (5)
N1—C5—C6—N2	−0.6 (4)	C25—C26—C27—C28	179.0 (3)
C4—C5—C6—N2	−179.8 (3)	C26—C27—C28—C29	2.5 (6)
N1—C5—C6—C7	179.1 (3)	C26—C27—C28—C34	177.6 (3)
C4—C5—C6—C7	−0.2 (5)	C27—C28—C29—C30	−3.4 (6)
N2—C6—C7—C8	0.5 (5)	C34—C28—C29—C30	−178.2 (4)
C5—C6—C7—C8	−179.1 (3)	C26—N6—C30—C29	1.5 (6)
C6—C7—C8—C9	0.3 (6)	C28—C29—C30—N6	1.4 (6)
C7—C8—C9—C10	−1.0 (6)	C32—O2—C31—O1	6.9 (5)
C6—N2—C10—C9	−0.3 (5)	C32—O2—C31—C23	−174.8 (3)
C8—C9—C10—N2	1.0 (6)	C22—C23—C31—O1	1.5 (5)
C15—N3—C11—C12	−0.1 (5)	C24—C23—C31—O1	−177.7 (3)
N3—C11—C12—C13	−1.6 (6)	C22—C23—C31—O2	−176.9 (3)
C11—C12—C13—C14	1.9 (6)	C24—C23—C31—O2	4.0 (5)
C12—C13—C14—C15	−0.5 (6)	C31—O2—C32—C33	179.9 (3)
C11—N3—C15—C14	1.6 (5)	C35—N7—C34—O3	−10.7 (7)
C11—N3—C15—C16	−177.9 (3)	C35—N7—C34—C28	166.9 (3)
C13—C14—C15—N3	−1.3 (5)	C27—C28—C34—O3	−13.6 (6)
C13—C14—C15—C16	178.1 (3)	C29—C28—C34—O3	161.2 (4)
C20—N4—C16—C17	−0.4 (5)	C27—C28—C34—N7	168.7 (3)
C20—N4—C16—C15	178.5 (3)	C29—C28—C34—N7	−16.4 (6)
N3—C15—C16—N4	−5.1 (4)	C34—N7—C35—C36	−18.7 (6)
C14—C15—C16—N4	175.5 (3)	C34—N7—C35—C40	165.0 (4)
N3—C15—C16—C17	173.8 (3)	C40—C35—C36—C37	1.5 (6)
C14—C15—C16—C17	−5.6 (6)	N7—C35—C36—C37	−174.7 (4)
N4—C16—C17—C18	0.3 (5)	C35—C36—C37—C38	−0.8 (6)
C15—C16—C17—C18	−178.5 (3)	C36—C37—C38—C39	−0.5 (6)
C16—C17—C18—C19	0.1 (6)	C36—C37—C38—C41	178.8 (4)
C17—C18—C19—C20	−0.4 (6)	C37—C38—C39—C40	1.0 (6)
C16—N4—C20—C19	0.1 (5)	C41—C38—C39—C40	−178.3 (4)
C18—C19—C20—N4	0.3 (6)	C38—C39—C40—C35	−0.2 (6)
C25—N5—C21—C22	3.7 (5)	C36—C35—C40—C39	−1.0 (6)
N5—C21—C22—C23	0.3 (5)	N7—C35—C40—C39	175.3 (3)
C21—C22—C23—C24	−2.9 (5)	C37—C38—C41—O4	7.6 (6)
C21—C22—C23—C31	177.9 (3)	C39—C38—C41—O4	−173.1 (4)
C22—C23—C24—C25	1.5 (5)	C37—C38—C41—N8	−171.6 (4)
C31—C23—C24—C25	−179.3 (3)	C39—C38—C41—N8	7.7 (6)
C21—N5—C25—C24	−5.2 (5)		

### Hydrogen-bond geometry (Å, °)

**Table:**

*D*—H···*A*	*D*—H	H···*A*	*D*···*A*	*D*—H···*A*
N7—H7···F2^i^	0.86	2.34	3.181 (4)	168
N8—H8B···F10^i^	0.86	2.29	2.999 (5)	139

Symmetry codes: (i) *x*, −*y*+1/2, *z*+1/2.
